# The Cognitive Architecture of Anxiety-Like Behavioral Inhibition

**DOI:** 10.1037/xhp0000282

**Published:** 2016-10-31

**Authors:** Dominik R. Bach

**Affiliations:** 1 Department of Psychiatry, Psychotherapy, and Psychosomatics and Neuroscience Centre Zurich, University of Zurich, and Wellcome Trust Centre for Neuroimaging, University College London

**Keywords:** approach/avoidance conflict, reinforcement learning theory, goal-comparator theory, behavioral inhibition, septo-hippocampal system

## Abstract

The combination of reward and potential threat is termed approach/avoidance conflict and elicits specific behaviors, including passive avoidance and behavioral inhibition (BI). Anxiety-relieving drugs reduce these behaviors, and a rich psychological literature has addressed how personality traits dominated by BI predispose for anxiety disorders. Yet, a formal understanding of the cognitive inference and planning processes underlying anxiety-like BI is lacking. Here, we present and empirically test such formalization in the terminology of reinforcement learning. We capitalize on a human computer game in which participants collect sequentially appearing monetary tokens while under threat of virtual “predation.” First, we demonstrate that humans modulate BI according to experienced consequences. This suggests an instrumental implementation of BI generation rather than a Pavlovian mechanism that is agnostic about action outcomes. Second, an internal model that would make BI adaptive is expressed in an independent task that involves no threat. The existence of such internal model is a necessary condition to conclude that BI is under model-based control. These findings relate a plethora of human and nonhuman observations on BI to reinforcement learning theory, and crucially constrain the quest for its neural implementation.

Situations that embody both reward and possible threat are common in many natural environments, and require an individual to trade off conflicting goals: avoiding threat and approaching rewards. Unsurprisingly, the study of such approach/avoidance conflict has a long history in experimental psychology (Miller, 1944). Across species boundaries and specific task designs, approach/avoidance conflict elicits particular behaviors (Aupperle, Sullivan, Melrose, Paulus, & Stein, 2011; Bach et al., 2014; Calhoon & Tye, 2015; Gray, 1982; Gray & McNaughton, 2000; Rodgers, Cao, Dalvi, & Holmes, 1997). This includes passive avoidance of the situation, behavioral inhibition (BI)—interruption of ongoing actions and suppression of overt approach or avoidance—and exploratory actions for risk assessment. Drugs that relieve human anxiety—anxiolytics—consistently attenuate these behaviors (Geller & Seifter, 1960; Pellow, Chopin, File, & Briley, 1985; Vogel, Beer, & Clody, 1971). Gray’s theory of a behavioral inhibition system (BIS) conceptualized these observations on a neuropsychological level (Gray, 1982; Gray & McNaughton, 2000). According to the BIS theory, interindividual differences in the workings of this system relate to anxiety disorders. This has inspired a large psychological literature on the relation of anxiety disorders and personality traits. Relevant traits are anxious temperament dominated by BI and assessed via behavioral observation in children (Fox & Pine, 2012) or nonhuman primates (Kalin & Shelton, 1989), and differential reinforcement sensitivity, assessed by self-report questionnaires in adults (Carver & White, 1994; Corr & Cooper, 2016). Beyond interindividual differences, however, the cognitive architecture of actual anxiety-like behaviors is still poorly understood. At the same time, a large neuroscience literature has focused on their neural implementation in nonhuman animals (Adhikari, Topiwala, & Gordon, 2010; Likhtik, Stujenske, Topiwala, Harris, & Gordon, 2014; McHugh, Deacon, Rawlins, & Bannerman, 2004). Crucially, and different from inhibitory control tasks, anxiety-like behavior requires cognitive inference on the statistics of the situation, for example, utilities and costs. Inhibitory control theory in contrast deals with situations in which clear instructions to act or to not act after certain sensory stimuli are given to a subject, and the task is to execute or withhold a quick motor action (Logan, 1981). Individual differences in such tasks are linked to trait impulsivity (Logan, Schachar, & Tannock, 1997) but not to anxiety.

Here, we provide a first attempt to characterize the cognitive architecture of human anxiety-like behavior in the terminology of reinforcement learning (RL) theory. In our analysis, we focus on the inference and planning process underlying BI, not on its execution. Experimentally, we capitalize on a recent translation of classical animal approach/avoidance conflict test to humans (Bach, 2015), in which we measure BI as a temporary suppression of approach responses, resulting in increased action latencies. This is based on previous work in nonhuman primates and rodents using operant conflict tests (Amemori & Graybiel, 2012; Friedman et al., 2015).

We have previously shown that in environments in which the occurrences of reward and threat are correlated in space and time (Prevedello, Dickman, Vieira, & Vieira, 2013; Sofaer, Sillett, Peluc, Morrison, & Ghalambor, 2013), anxiety-like BI is adaptive in a decision-theoretic sense—it maximizes the expected overall outcome of a situation (Bach, 2015). This is in line with Gray’s proposal that the BIS serves an adaptive function (Gray, 1982; Gray & McNaughton, 2000). Furthermore, we demonstrated that the pattern of human BI under different levels of threat probability and threat magnitude replicates the theoretically adaptive pattern in such an environment (Bach, 2015). This did however not address how BI is controlled from the perspective of an agent. BI may well appear adaptive for an external observer, but still the agent may not know or evaluate this.

Hence, in the current study, we first addressed whether BI happens to be adaptive in particular environments (something that may possibly have favored its evolution), or whether this adaptiveness is also evaluated by the agent (Gray & McNaughton, 2000). In the terminology of RL theory and cognitive psychology, such distinction can be related to the difference between “Pavlovian” and “instrumental” mechanisms (Dayan & Balleine, 2002; Dayan & Berridge, 2014; Dickinson & Balleine, 1994). Pavlovian actions are tied to particular eliciting stimuli, independent of their actual consequences. This may be adaptive if the actual consequences are favorable most of the time in the natural habitat of the organism. In contrast, instrumental actions are selected by the agent to yield the most favorable outcome in a specific situation. Pavlovian actions do not change if the action/outcome contingencies become unfavorable in experimental circumstances, a phenomenon termed “negative automaintenance” in the RL literature (Schwartz & Williams, 1972). In contrast, instrumental actions adapt to changes in action/outcome contingencies (Dickinson & Balleine, 1994). The fact that in natural environments, rodent anxiety-like behavior can change over time (Fonio, Benjamini, & Golani, 2009) may suggest that it is at least partly under instrumental control. This motivates our hypothesis in Experiment 1 that human BI would at least gradually attenuate when action/outcome contingencies are unfavorable, that is, when the degree of BI negatively influences the expected outcome.

If anxiety-like BI is under instrumental control then a second and related question is how the outcomes are evaluated. Two general classes of cognitive algorithms have shown to exist in humans, to solve different problems (Daw, Niv, & Dayan, 2005). Model-based reasoning requires a detailed model of the environment, enabling a prospective and explicit (although not necessarily conscious) evaluation of all possible future outcomes and their probabilities; action is then selected to maximize the expected future outcome (Daw et al., 2005; Dickinson & Balleine, 1994). This algorithm allows fast adaptation when changes in the environment are detected because the model can be altered almost instantaneously. However, it presumably requires computation time and memory resources. In contrast, model-free control corresponds to RL algorithms (Mackintosh, 1975; Pearce & Hall, 1980; Rescorla & Wagner, 1972) in which the value of possible actions in a given situation is learned retrospectively after outcomes are experienced, without prospective evaluation of future outcomes (Sutton & Barto, 1998). In any given state, the agent then chooses the action that has in the recent past maximized the expected future outcome. Such algorithms are simple and do not require many resources in terms of time or working memory. But because learning takes place gradually and retrospectively, an agent using such control mode cannot quickly adapt to local changes in the environment. We have previously demonstrated that human BI is consistent with the use of explicit Bayesian models that incorporate subjective assumptions on threat/reward correlations (Bach, 2015), which may suggest model-based cognitive control. However, this did not prove that humans actually computed such models. Indeed, simple model-free heuristics can often be used to approximate model-based reasoning (Gigerenzer & Gaissmaier, 2011). Here, we speculated that if humans use a model-based strategy to control anxiety-like BI, it should be possible to access the model they use even in an independent task, not involving any threat (Houlsby et al., 2013). Hence, we created a safe predator exposure task for Experiment 2. We hypothesized that in this task, humans express subjective beliefs on threat/reward correlations of the sort that would make anxiety-like BI adaptive in an approach/avoidance task.

## Experiment 1

This experiment addressed the question whether anxiety-like BI is reduced when it leads to negative consequences, and therefore, whether it is under Pavlovian or instrumental control. To this end, we adapted a previously developed operant approach/avoidance conflict paradigm, embedded in a “scoop-and-run” computer game (Bach, 2015). This task affords analysis of individual actions, and thus differs from our previous “stay-and-play” approach/avoidance conflict task (Bach et al., 2014). One group played the game in a version in which BI had no influence on threat probability, as in our previous work. In this version, participants started each trial in a safe place where the predator could not reach them. In the second group, participants would start outside the safe place. The predator could thus catch them if they hesitated to make a response; thus, BI would increase threat probability. We hypothesized that participants in Group 2 would show reduced BI after experiencing the unfavorable consequences. We expected to find this both in an overall group difference, and also in a Group × Time interaction, that is, participants in Group 2 would reduce approach latency over time. A between-subjects design was used because behavior in approach/avoidance conflict tasks has been described to become stereotypical with extended practice (Gray & McNaughton, 2000).

### Method

#### Participants

We recruited 38 participants from the student and general population (19 female, mean age ± standard deviation: 23.7 ± 3.7 years), and assigned them to two gender-balanced groups. The sample was independent from Experiment 2 and from a previous study using the same setup (Bach, 2015). State anxiety values for all but one participant, and trait anxiety for all but three participants, were within 2 standard deviations around the reference sample mean (586 individuals between 15- and 29-years-old, both sexes; Laux, Glanzmann, Schaffner, & Spielberger, 1981). State anxiety values were comparable to the reference sample (35.07 vs. 36.8, *p* = .13, Welch’s *t* test) and trait anxiety values slightly higher than the reference sample (39.25 vs. 35.1, *p* = .002). All participants gave written informed consent after being fully informed about the purpose of the study. The study protocol, participant information, and form of consent, were in full accordance with the Declaration of Helsinki and approved by the competent research ethics committee (Kantonale Ethikkommission Zurich).

#### Design and procedure

**Figure 1 fig1:**
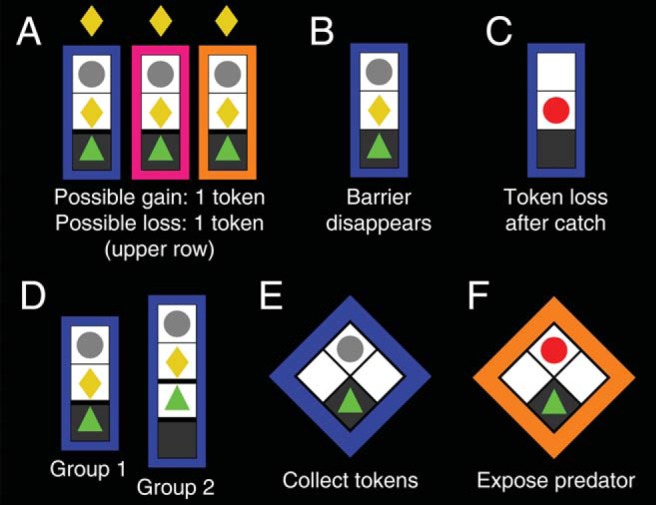
Experimental setup. (A) In Experiment 1 a human player (green triangle) rests in a safe place on the bottom of grid, while a “predator” is sleeping at the top (gray circle). On each epoch, six successive reward tokens (yellow rhombi) appear. The colored frame indicates the threat level of the sleeping predator with color/threat association balanced across subjects. (B) Tokens are separated from the player by barriers that disappear at a random time point. Once they have disappeared, the time until the token is removed is exponentially distributed. The player can press a key to collect the tokens, and thus accumulate up to six tokens over any given epoch. At any time during the epoch, the predator becomes active with equal probability, but once active it will only reveal itself if the player is currently outside the safe place and outside barriers; the predator can never reach the safe place or cross the barriers. (C) If the player is caught by the predator, it loses all tokens already collected in this epoch, and no more new tokens appear. Magnitude of potential loss therefore corresponds to the number of already collected tokens. Threat level is defined as the wake-up rate, which was different for the three predators. (D) In Group 1, the starting safe is protected from the predator. For Group 2, starting place is outside the safe place. Participants played 270 epochs, thus making up to 1,620 choices. (E) In Experiment 2, participants played the same approach/avoidance Task 1 on a 2 × 2 grid. (F) Following approach/avoidance Task 1, participants in Experiment 2 were instructed to press a key to “expose” the status of the predator in safe predator exposure Task 2. See the online article for the color version of this figure.

The experiment followed a 2 × 3 × 6 factorial design with the between-subjects factor starting place (inside/outside safe place) and the within-subjects factors threat level (low/medium/high) and possible loss (0–5 tokens). Threat level corresponds to wake-up rate of the predator, and thus, to loss probability. Potential loss corresponds to number of already collected tokens which would be lost if the player got caught. A modified version of a previously developed approach/avoidance computer game (Bach, 2015) was presented on a standard LCD monitor (see Figure 1) in six blocks of 45 epochs. In each epoch, a sequence of six reward tokens appeared at random time points; the player could decide each time whether or not to approach and collect the token. The primary dependent measure was approach latency on those trials on which participants chose to approach. Participants received a fixed payment and an additional reward for the number of retained tokens of one randomly drawn epoch at the end of the experiment. A “sleeping predator” was waiting above the token and could become active with a probability that was constant over time. Once active, if the human player was inside the safe place, it deactivated itself. If the human player was outside the safe place (regardless whether or not a reward token was present), it revealed itself and moved to the human player’s grid block. The human player was “eaten” and all previously collected reward tokens from this epoch were removed. Once the predator was active, the human player had no possibility to escape if he or she was in the neighboring grid block. In order to remove any time benefit (opportunity cost) from getting caught by the predator, the active predator staid visible on the screen for the remaining time of the epoch while the human player had to wait.[Fig-anchor fig1]

#### Stimuli and task statistics

The game was presented on a 1 × 4 grid in vertical orientation (∼1.5° horizontal visual angle). The player was placed on the starting position, confined by one (Group 1) or two (Group 2) “barriers” to prevent the player from moving. Starting position was in the safe place for Group 1, and in the grid block above the safe place for Group 2. A token was visible in the grid block above the player.

After a variable interval drawn from an exponential distribution with a mean of 1.25 s, the barriers were removed, and the player was free to move and collect the token, under risk of getting caught. Note that the presence of barriers, and of the token in the delay period, is different from Experiment 2 and from a previous report (Bach, 2015). The reason for making the token visible in the delay period in this version of the game was to simplify the graphical setup such that only one visual event (barrier removal) occurred to signal the possibility for token collection. The interval during which the reward token was present and could be collected was also drawn from an exponential distribution with a mean of 1.25 s. If not collected, the token disappeared at the end of this interval. Whether or not it was collected, the player was moved to the starting position 250 ms after the token disappeared, and the barrier above the player put in place. The next trial within the epoch started 250 ms later. The wake-events of the predator followed a homogenous Poisson process, independently determined in successive time bins of 20 ms duration. The wake-up rate was set such that the probability of getting caught was p_1_ = 0.1, p_2_ = 0.2, and p_3_ = 0.3, respectively for the three threat levels, if the player stayed outside the safe place for 100 ms (Group 1) or 600 ms (Group 2). These latency values approximated values found in previous experiments. Thus, the event rate for the three threat levels was, respectively, λ_1_ = 1.0536, λ_2_ = 2.2314, and λ_3_ = 3.5667 for Group 1, and was divided by 6 for Group 2. Actual catch rates depend on participants’ response latencies and turned out to be 0.08/0.18/0.25 for Group 1, 0.05/0.10/0.17 for Group 2 when making an approach response, and 0.02/0.03/0.04 for Group 2 when making an escape response. The human player was controlled with the up/down cursor keys on a standard computer keyboard. The player could move between grid blocks at all times unless restricted by barriers or caught by the predator, but it could never reach the top grid block occupied by the sleeping predator.

#### Data analysis

All data are necessarily unbalanced because the number of data points for each cell in the design depends on behavioral choices and on chance.

When the participant approached the token, we extracted the approach latency as main dependent variable. We also analyzed return latencies, that is, time passed between approaching the token and moving back. In Group 2 the player had to make two movements to go to the safe place; hence there are two return latencies. For escape choices in Group 2 we extracted escape latency. To avoid response latencies being biased by extreme values, they were only analyzed if they fell into response windows of 150 ms < approach/escape latency < 2,000 ms and 0 ms < return latency < 2,000 ms, as in a previous study (Bach, 2015). This excluded, in Group 1, 1.7% of approach latencies and 3.5% of return latencies. In Group 2, this excluded 4.5% of approach latencies, 5.7% of escape latencies, and 1.5% or 7.6% of the first or second return latencies, respectively. Choices were reconstructed by creating six data points for each epoch, corresponding to the possibility of collecting six tokens. For each of these six tokens, we recorded 1 if the individual chose to collect up to, or more than, this number of tokens on this epoch, and 0 if the individual chose to collect less than this number of tokens on this epoch. Choices in epochs on which the player was caught cannot be reconstructed and were therefore not analyzed. The resulting data are serially correlated by design. Most players rarely collected the sixth token such that some design cells were empty and the parameters could not be estimated reliably. Therefore, the sixth token was excluded for all reaction time (RT) analysis. The resulting model followed a 2 (Group) × 3 (Threat Level) × 5 (Potential Loss) factorial design, and for choice data a 2 × 3 × 6 design. To analyze changes in approach latency over time, we split the data into the 6 blocks and added the main effect of block and the Block × Group interaction to the model. Finally, in addition to the full factorial model, we also analyzed data from both groups separately in 3 × 5 or 3 × 6 factorial models, in order to facilitate comparison with previous publications.

The *lme4* package in the software R (www.r-project.org) was used for all inference statistics as it provides meaningful parameter estimators for unbalanced data sets. Choice data were analyzed using a generalized linear mixed effects model (glmer) for binomial data, and RTs in linear mixed effects models (lmer). We did not transform RTs, as we had no a priori reason to do so and a previous report demonstrated that analysis of transformed RTs replicates analysis of raw RTs (Bach, 2015). All models included a random subject intercept. Fixed-effects *F*-statistics were extracted using unpartitioned error variance and the R function ANOVA; *p* values were calculated by using a (conservative) lower bound on the effective denominator degrees of freedom as *df* = *N* − K, where *N* is the number of observations, and K is the number of all modeled fixed and random effect parameters. No *p* values were computed for the choice data as they are autocorrelated across the “potential loss” factor by construction, and therefore have reduced effective numerator degrees of freedom. Mean RTs were reconstructed from the linear mixed effects model using the function lsmeans. In a nutshell, this function averages the data for each subject and experimental condition separately, and then averages over subjects, while correcting for missing values in individual subjects.

### Results

**Figure 2 fig2:**
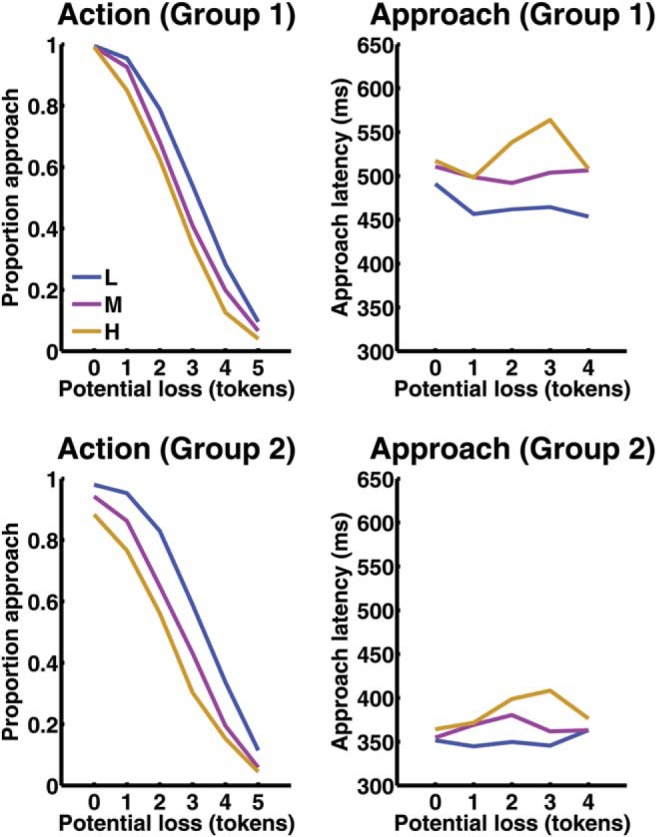
Results from Experiment 1. The graphs show responses to the possibility of collecting the nth token after already having collected (*n* – 1) tokens, which constitutes the potential loss. L = low threat; M = medium threat; H = high threat; Action = Proportion of epochs in which the player chose to collect at least the nth token. Because the players rarely approached after collecting five tokens, approach latency is only shown up to a potential loss of four tokens. As the data are unbalanced, mean approach latencies were estimated in a linear mixed effects model with random intercepts. Approach latency is markedly shorter in Group 2 than Group 1. See the online article for the color version of this figure.

Approach latency in Group 2 was around 150 ms shorter than in Group 1, a highly significant difference (Figure 2, Table 1). While threat level increased approach latencies in both groups, this influence was smaller in Group 2, as indicated by a significant Group × Threat Level interaction. Additionally, the effect of potential loss on approach latencies was different between the groups, and there was a significant three-way interaction. Next, we analyzed how BI developed over time, in both groups, by splitting the data into six blocks of 45 epochs. We found a significant Group × Block interaction (see Results in supplemental materials). Approach latency was reduced between Blocks 1–2 from 515 ms to 507 ms in Group 1, and from 404 ms to 358 ms in Group 2. For comparison with a previous report we analyzed Group 1 separately. Threat level, potential loss and their interaction, influenced approach latencies with a similar pattern as in previous reports, but there was no linear effect of potential loss (Table 1, Figure 2, see Results in supplemental materials).[Fig-anchor fig2][Table-anchor tbl1]

### Discussion

We asked whether BI is reduced when it increases threat probability. We found that in this case, approach latencies were about 150 ms shorter than in a control group in which BI had no impact on threat probability. This suggests that BI adapts to unfavorable consequences. Alternatively, this overall group difference could be explained if participants used a Pavlovian, but model-based strategy (Dayan & Berridge, 2014) to precompute their behavior even before the experiment started, rather than instrumentally learn from their actions. In other words, according to this explanation they would not take into account consequences of BI, but respond to the Pavlovian cues of being inside or outside the safe place when they started. To exclude such possibility, we showed that participants in Group 2 adapted their behavior over time to a greater degree than in control Group 1. In particular, we observed a pronounced reduction in approach latencies from Block 1 to Block 2 in Group 2 (46 ms) but not Group 1 (8 ms). Furthermore, we note that in spatial approach/avoidance conflict tasks, anxiety-like BI is elicited also outside safe compartments in rodents (Fonio et al., 2009) and humans (Bach et al., 2014). Thus it appears unlikely that in the current task, BI should depend on the qualitative aspect of being in a protected starting position. Finally, the starting place in Group 2 was quantitatively no less safe than in Group 1: In case of an escape response in Group 2, participants were rarely caught, just as when making no response in Group 1. In case of an approach response, participants in Group 2 adapted their approach latency to an extent that overall, they were caught less often than in Group 1. All in all, it appears that instrumental consequences of BI lead to its reduction, rather than Pavlovian cues.

Behavior in the control group was comparable to a previous report (Bach, 2015), underlining the validity of the modified experimental setup. Different from the previous report and from Experiment 2, however, we observed that the influence of possible loss on approach latency was not linear. A possible explanation is that the token was already visible before the participant could make a movement. According to our previous model, BI arises from subjective assumptions on threat/reward correlations, corresponding to a situation in which a predator is alerted by the occurrence of his prey’s reward. The influence of possible loss on approach latency in this model depends on the curvature (second derivative) of the temporal evolution of threat probability. It appears possible that the temporal evolution of subjective threat probability after a reward occurs is different from the evolution after a barrier is removed, and this would lead to a different impact of possible loss.

## Experiment 2

After having shown that anxiety-like BI is likely under instrumental control, we asked whether it is based on an explicit (although not necessarily conscious) model of the environment. Experiment 2 therefore addressed whether possible assumptions about threat/reward correlations in the approach/avoidance task are explicitly expressed in a different task, not involving any threat and thus not involving BI. Such threat/reward correlations exist in natural environments (Prevedello et al., 2013; Sofaer et al., 2013) but they are objectively absent from our task. However, we have previously shown that anxiety-like BI would be adaptive from the perspective of an agent if the agent subjectively assumed such correlations. Experiment 2 had two tasks. In approach/avoidance Task 1, participants were familiarized with the computer paradigm and collected tokens. Next, they engaged in safe predator exposure Task 2. Here, they were could expose the status of the predator by key press, without threat of getting caught. They were rewarded if they exposed the predator just at the moment when it was awake. Under a null hypothesis that participants had no assumptions on threat/reward correlations, the timing of their exposure attempts should be independent from the occurrence of incidental and unobtainable tokens. We hypothesized that such assumptions exist, and that exposure attempts would be more frequent immediately after tokens.

### Method

#### Participants

We recruited 20 participants from the student and general population (10 female, mean age ± standard deviation: 23.6 ± 3.7 years). The sample did not overlap with Experiment 1 or a previous report (Bach, 2015). State anxiety values for all and trait anxiety for all but two participants, were within 2 standard deviations around the reference sample mean (Laux et al., 1981). State anxiety values were slightly lower (33.4 vs. 36.8, *p* = .04), and trait anxiety values slightly higher than the reference sample (38.6 vs. 35.1, *p* = .05). All participants gave written informed consent after being fully informed about the purpose of the study. The study protocol, participant information, and form of consent, were in full accordance with the Declaration of Helsinki and approved by the competent research ethics committee (Kantonale Ethikkommission Zurich).

#### Design and procedure: Approach/avoidance Task 1

This part realized a 3 × 6 factorial design with the within-subjects factors threat level (low/medium/high) and possible loss (0–5 tokens). Participants played four blocks (Blocks 1–2, 5–6) of 45 successive epochs of the previously reported computer game (Bach, 2015). The game was the same as in Group 1 of Experiment 1, with the only difference that the playing field was a 2 × 2 grid in diamond orientation (∼4.0° horizontal angle), there were no barriers, and the timing was therefore slightly different. Specifically, at the start of each epoch, the player was in a safe place in the bottom grid block. A token could appear either to the left or to the right. The sleeping predator was waiting in the top grid block. As there were no barriers, the player was free to move during the entire epoch unless caught be the predator. The interval during which the reward token was present and could be collected was drawn from an exponential distribution with a mean of 1.25 s. If not collected, the token disappeared at the end of this interval. After this, whether or not the token was collected, a waiting interval started that lasted 500 ms plus a random sample from an exponential distribution with mean of 1.25 s, before the next token came on the screen or the epoch ended. The predator wake-up rates were the same as for Group 1 in Experiment 1. The human player was controlled with the left/right cursor keys on a standard computer keyboard.

#### Design and procedure: Safe predator exposure Task 2

In Blocks 3–4, participants were given a different task on 36 epochs per block, randomly interspersed with nine epochs of approach/avoidance Task 1. The type of task was graphically signaled (gray rhombus or gray circle under the grid). The graphical setup of Task 2 was exactly the same as in Task 1, but participants could not move on the grid and always stayed in the safe place. They were tasked to “expose” the awake predator by pressing the cursor up key. If the predator was awake at this point in time, it would turn red, and the next epoch would start. This provided an incentive for speeded responses. If the predator was sleeping, it would turn black for 100 ms and the epoch would continue. This feedback gave participants an opportunity to learn the experimental statistics, according to which the probability of being awake was independent of time, or of token appearance. On each epoch, the human player had 6 attempts to expose the predator, after which the key was disabled until the epoch ended. Participants were explicitly informed that the tokens were irrelevant to the task. One randomly selected epoch from Task 2 was rewarded at the end of the experiment; if the participant successfully exposed the predator he or she gained as much as from collecting two tokens in Task 1. Under this 1/0 loss function, the reward-maximizing strategy is to press the key at the moment when the participant thinks the predator is most likely to be awake, that is, at the maximum of the temporal evolution function of the predator wake-up probability. The objective wake-up probabilities of the three predators were p_1_ = 0.1, p_2_ = 0.2, p_3_ = 0.3, and constant over time. Whether the predator was awake or asleep was randomly determined each time the participant made an exposure attempt. If the participant (correctly) assumes that this probability is constant over the epoch, then from his or her perspective, reward does not depend on the timing of key presses, there is no need to preferentially press the key after incidental tokens occur, and key presses could be uniformly distributed across the epoch. In fact, all else being equal, if participants wish to shorten the experiment, then an optimal strategy would be to press the key immediately after the epoch has started. In both cases there would be no dependency of key presses on token appearance. If participants however, assumed temporal threat-reward correlations, the reward-maximizing strategy is to press a key immediately after an incidental token has occurred, at the maximum of their subjective threat evolution function.

#### Data analysis

For approach/avoidance Task 1, the data analysis was the same as for the control Group 1 in Experiment 1. We additionally analyzed correctness of response (left/right). For the safe predator exposure Task 2, we sought to determine whether participants’ responses depended on the appearance of irrelevant tokens. To this end, we split the data into key presses made before the first token appeared, and those made later. For key presses after the first token, we computed the latency of each response with respect to the most recent token that preceded it, and analyzed the ensuing RT distributions. The distribution of these responses was compared against two null distributions with a Kolmogoroff-Smirnoff (KS) test. A list of variables and symbols used in the derivation of these null distributions is included in Method section of supplemental materials.

Crucially, a key press at time *T* after appearance of a particular token will only be assigned to that token if the next token has not yet appeared at time *T*: *0* < *T* < *T*_*2*_, where *T*_*2*_ is the interval between a token and the next one. If *T* > T_*2*_, the key press would be assigned to the next token. To give an intuition, because the next token becomes more likely to appear as time passes, we are more likely to observe a key presses early than late after a token, even under the null hypothesis that they are independent from token appearance. Hence, it is necessary to quantitatively specify the RT distribution under the null hypothesis. Because key press and appearance of the next token are independent events, $p_{T=t,T_{2}>t}(t),$ the probability density of observing a key press at time *t*, is

$$p_{T=t,T_{2}>t}\left(t\right)=p_{T=t}\left(t\right)p_{T_{2}>t}\left(t\right),$$

and the probability of observing any key press after a token is

$$Pr\left[O=1\right]=\int_{0}^{\infty }p_{T=t}\left(t\right)p_{T_{2}>t}\left(t\right)dt.$$

Hence, the probability density of observed key presses after a token has appeared is

$$p_{T=t,T_{2}>t|\text{O=1}}\left(t\right)=\frac{p_{T=t}\left(t\right)p_{T_{2}>t}\left(t\right)}{\int_{0}^{\infty }p_{T=t}\left(t\right)p_{T_{2}>t}\left(t\right)dt}.$$ 1.1

If participants distribute their responses uniformly across an epoch, then $p_{T=t}\left(t\right)=c,\;c\in {\mathbb{R}}^{+},$ and the above equation reduces to

$$p_{T=t,T_{2}>t|\text{O=1}}\left(t\right)=\frac{c\;p_{T_{2}>t}\left(t\right)}{\int_{0}^{\infty }c\;p_{T_{2}>t}\left(t\right)dt}=p_{T_{2}>t}\left(t\right).$$

In other words, under the null hypothesis, observed responses after a token appeared are random observations from a variable distributed according to $p_{T_{2}>t}(t).$ This distribution is given by the experimental set up and was expressed analytically (see supplemental methods for details).

However, participants may distribute their key presses unevenly across the epoch, but these may still be independent from token appearance. For example, participants may be more likely to press early in an epoch and exhaust their allotment of key presses. We used equation (1.1) to simulate observations from the null distribution in this case. We first gathered all RT expressed wrt. to epoch start, across all epochs and participants. Then, we simulated the distribution of RT wrt. token appearance. We went through all token appearances throughout all epochs, and added to the distribution all key presses that occurred later in the epoch than this token appearance, expressed wrt. this token appearance. This approximation to $p_{T=t}\left(t\right)$ was then combined with the analytically derived $p_{T_{2}>t}(t),$ by drawing for each observed value of *T* in the RT distribution a random observation from $p_{T_{2}>t}\left(t\right)$ which could be true or false, and retaining the RT value only if it was true. Because of the large number of data points in the RT distribution (>1e8), this simulation procedure was performed only once.

**Figure 3 fig3:**
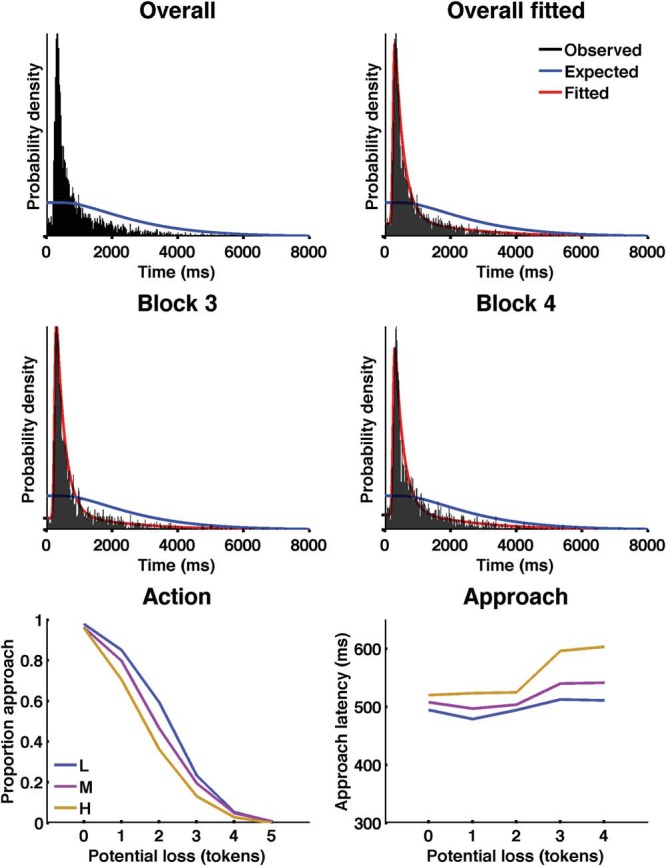
Results from Experiment 2. Top panels: RT distributions for Task 2 in Experiment 2. RT are expressed with respect to the token appearance that preceded the response. Blue lines: RT distribution expected under the null hypothesis. Red line: Fit with the winning model, a combination of an ex-Gauss model with the null distribution. Because the inter-token-interval is a random variable, responses are less likely to be observed at long latencies than at shorter latencies, even under the null hypotheses. However, responses are much more frequent than expected directly after a token has appeared. Middle panels show RT distributions split up between two subsequent blocks. Y-Ticks = Estimated proportion of responses fit by the null distribution in the combined model. Bottom panels: Results from approach/avoidance Task 1. See the online article for the color version of this figure.

We could thus compute a one-sample KS test of observed RT against an analytical null distribution (under the assumption that RTs are uniformly distributed over an epoch) and a two-sample KS test of observed RT against the null distribution of simulated RT (under the assumption that RT are nonuniformly distributed across the epoch but independent of token appearance). KS tests were computed in R. The null distribution shown in Figure 3 is the analytical distribution; the simulated distribution looks similar.[Fig-anchor fig3]

Finally, a difference between the two blocks of the task was assessed in a two-sample *t* test of mean RTs for each participant and block, and in a two-sample KS-test of all responses from each block.

#### Reaction time models

Under the null hypothesis, participants would distribute their key presses independent from token appearance (Model 1). If, on the other hand, participants assume the predator is more likely to wake up directly after a token occurs, and therefore press a key as quickly as possible after a token, this amounts to a simple RT task. RTs in such tasks can well be described by an exponential Gaussian (exGauss) model, a convolution of an exponential with a Gaussian distribution (Hohle, 1965; Model 2). However, some key presses were made very early after a token such that they must have been initiated before, and a small number of responses was made even before the first token appeared. Hence, a simple RT model may not capture the full RT distribution. We therefore speculated that two different psychological processes could generate responses on different subsets of trials: first, a process distributing responses uniformly across the epoch that could be modeled by our null distribution. The second process would be described by a simple RT Model 2. The relative weight of the two processes was fitted to the RT distributions (Model 3). Finally, to capture differences between the two task blocks, individual parameters were either fitted across both blocks, or for each block individually. To formally summarize, we fitted the RT distribution with the following models:

1 Null model: the null distribution based on a uniform distribution of key presses across the epoch (0 parameters).
2 Simple RT model: an exponential Gaussian distribution (three parameters), describing the sum of a exponentially and a normally distributed random variable, which has the following pdf:
  $$p_{exGauss}\left(t\right)=\frac{\lambda_{ex}}{2}e^{\left(\lambda_{ex}/2\right)(2\mu +\lambda_{ex}\sigma^{2}-2t)}erfc\left(\mu +\lambda_{ex}\sigma^{2}-t/\left(\sigma \sqrt{2}\right)\right)$$
3 Combined RT and null model: a weighted sum of null distribution and RT model (four parameters) with the following pdf, and 0 ≤ w ≤ 1:
  $$p_{com}=wp_{exGauss}\left(t\right)+\left(1-w\right)p_{T_{2}>t}\left(t\right)$$
4 Model 3 with block-specific parameters for λ, μ, σ, or w (five parameters).

Model parameters and likelihood were estimated using the in-built Matlab function mle.m. We quantified model evidence as Bayesian Information Criterion (Raftery, 1995) and considered an absolute BIC difference >3 as significant, in analogy to classical *p* values (Burnham & Anderson, 2004; Penny, Stephan, Mechelli, & Friston, 2004). We repeated the procedure under three different assumptions: (a) that the RT distribution and its parameters are the same for all participants (fixed-effects parameters); (b) that the RT distribution is the same for all participants but the parameters are not (random-effects parameters); and (c) that the RT distribution and its parameters were different for each participant (random-effects model structure). Each analysis can lead to a different result, but in fact they all converged in the current data set such that the interpretation is unambiguous. Under assumption (a), the RT distribution was fitted to all data from all participants; under assumption (b), the RT distribution was fitted to data from each participant individually, and BIC values added up; and under assumption (c), the participant’s individual BIC scores were entered into a group level random effects analysis (Stephan, Penny, Daunizeau, Moran, & Friston, 2009).

### Results

When instructed to collect tokens in approach/avoidance Task 1, participants behaved similar to previous reports and similar to Experiment 1 (Figure 3, Table 2). Figure 3 shows participants’ responses in the safe predator exposure Task 2. Based on the Bernoulli probability that the robber was awake upon an exposure attempt, in which case the epoch would end, we expected participants to make on average 271.6 exposure attempts. We observed 243.15 ± 42.8 (mean ± *SD*) responses per participant. Most (93.2%) of these exposure attempts were made after a first token had appeared. These were expressed with respect to the time point of the preceding token. This analysis revealed that responses were made preferentially just after a token had appeared on the screen (see Figure 3). The RT distribution was tested against a null hypothesis that responses are independent from token appearance. The difference was highly significant (*p* < .001) in both KS-tests, showing that participants were more likely to make a response after a token had appeared than otherwise. [Table-anchor tbl2]

Next, we were interested in the psychological mechanism generating the RT distribution in safe predator exposure Task 2. Across both task blocks, a model combining an exGauss distribution with the null distribution had significantly higher evidence (smaller BIC values) than the null model (BIC_com_ − BIC_null_ = −4,766) or a simple exGauss model (BIC_com_ − BIC_exGauss_ = −1,062), despite penalizing increased model complexity (see Figure 3). Across the group, individual participant data were also best fit by this model (BIC_com_ − BIC_null_ = −5,761; BIC_com_ − BIC_exGauss_ = −1,162). We noted that two persons had a qualitatively different RT distribution (see Results in supplemental materials) such that it may be justified to regard model structure as a random effect. We estimated in a random effects analysis the proportion of the population for which the RT distribution followed the null, exGauss, and combined model. These were 4.3%, 17.2%, and 78.4%, respectively. The probability that the combined model is more frequent in the population than the other two models (exceedance probability) was p_x_ = .9993. In sum, the combined model provided the best fit both on the group level and for the individual participants’ data.

Finally, we sought to investigate whether participants adapted their behavior over time, because they were given feedback on the predator status and could therefore learn that there was in fact no relation of threat and reward. Between Blocks 3 and 4, mean RT across all participants significantly increased (926.9 vs. 999.3 ms, *t*(4,838) = 2.5, *p* = .02), and we found a significant difference between RT distributions (KS-test, *p* < .001). Hence, we sought to determine the underlying psychological process generating this difference. We compared, on the group level, the combined model containing parameters across both blocks, with models that split up one parameter between the two blocks. We found highest evidence for a model with block-specific parameters for the parameter *w*, the ratio of responses controlled by the two processes. Parameters from this model suggested that participants changed the ratio of token-independent responses from 35% (Block 3) to 41% (Block 4; Figure 3, y-ticks in middle panels). In keeping with this, we descriptively also observed a small increase in responses made before any token occurred (6.4% vs. 7.2%).

### Discussion

In this experiment, we probed whether humans express subjective beliefs on threat/reward correlations in approach/avoidance conflict task, by giving them a different task not involving actual threat. We asked them to press a key when they thought a virtual predator was awake and found that participants were more likely to make a response just after an incidental and unobtainable reward token had appeared on the screen. This is in keeping with a hypothesis that they believe a threat is more likely at a time when a potential reward has just occurred. Such beliefs would make anxiety-like BI in the approach/avoidance task optimal from the agent’s perspective: BI would then maximize overall expected utility (Bach, 2015).

RT distributions in the task were best described by the combination of an exGauss model, which usefully describes RTs in many simple reaction tasks, with the null distribution. According to this combined model, some key presses are responses to token appearance, and others are indeed randomly distributed across an epoch. One may speculate that the latter responses serve the goal of exploring the statistics of the task, which is a meaningful strategy even under subjective beliefs on threat/reward correlations. Crucially, these different processes of response generation did not relate to different participants, but occurred within the same persons, because the combined model provided the best fit even on an individual subject level. A random-effects analysis revealed that for a small proportion of the population, RT distributions can better be described by a single process: either a purely token-independent process (<5%) or a purely token-dependent process (<20%). For most individuals (>75%), both processes occur on different trials.

We gave feedback on the predator status whenever participants made a response. This gave them an opportunity to learn that objectively there were no threat/reward correlations in our task, and that their response timing made no difference to the success of exposing the predator. We found that participants changed their behavior between two task blocks into the direction of more token-independent responses. However, the majority of key presses were still made in response to incidental tokens.

Finally, we note that behavior in the approach/avoidance Task 1 was similar to a previous report with the same setup (Bach, 2015). In particular, and different from Experiment 1, we observed a linear effect of threat probability and of possible loss on approach latencies.

## General Discussion

Anxiety-like behavioral inhibition is commonly observed in experimental approach/avoidance conflict tasks that build on the coexistence of reward and threat. We have previously shown that this can be adaptive from an external observer’s perspective in many natural environments (Bach, 2015). The current study addressed how this behavior is controlled from the perspective of the agent. Results from Experiment 1 suggest that BI is at least partly instrumental—it is reduced when it becomes maladaptive and leads to increased threat probability. This is in keeping with our previous suggestion that the adaptive function of BI is to reduce threat under environmental conditions in which threat and reward are correlated in space and time (Prevedello et al., 2013; Sofaer et al., 2013). Results from Experiment 2 suggest that agents subjectively also assume such correlations in our paradigm. Specifically, we show that in an independent safe predator exposure task, embedded in the paradigm but not involving any threat, behavior is consistent with the existence of such subjective assumptions. While we cannot prove that these assumptions are also used to control BI, the existence of these assumptions constitutes a necessary condition to conclude that anxiety-like BI is under model-based control, something that is often equated with goal-directed behavior (Daw et al., 2005). The goal, in our case, would be to avoid a threat encounter, something that BI helps to reduce under the internal subjective model.

A sufficient criterion for goal-directed control, put forward by Dickinson and Balleine (1994) in the context of reward-based decision making, is a precise representation of the goal, something that was not addressed in the current study. This can in principle be tested by reinforcer devaluation—changing the desirability of the goal. Typical experiments of this sort use a particular reward which is made undesirable by the use of homeostatic principles; for example, food is becoming less attractive by satiation. In our case, the ultimate goal of avoiding threat encounter appears more difficult to devaluate although perhaps not entirely impossible. For example, during foraging for food, there may be states of resource depletion in which charging a predator aggressively entails higher survival chances than avoidance of the encounter, because passive avoidance would likely lead to starvation. This is a possibility that one could test in future experiments, for example building on virtual foraging scenarios in humans (Korn & Bach, 2015).

Objectively, there are no threat/reward correlations in our approach/avoidance task. However, a player assuming such correlations has little chance to learn this. This is because the player would have to make early approach responses to find out that the threat probability is constant over time—but from the subjective perspective, such early responses would expose the player to harm. The safe predator exposure task was designed to allow such exploration. First, there was no explicit threat regardless of the predator’s status. Second, unsuccessful exposure attempts had no negative consequences: participants could make six exposure attempts per epoch, but only one successful exposure could be rewarded. This made it possible to perform exploratory actions and find out the objective statistics of the task. Indeed, the RT model that best fit our data combined a majority of responses immediately after tokens with a smaller proportion of responses made independent of tokens. The former are expected under subjectively assumed threat/reward correlations, while the latter could possibly serve exploration. Between the two task blocks, participants increased the proportion of key presses unrelated to the tokens. However, we also note that despite extensive training, the majority of responses was still made immediately after tokens. This may imply that participants start the task with precise prior assumptions which require many additional observations to become properly adjusted to objective task statistics.

We addressed the inference and planning process behind anxiety-like BI, not its motor execution. That is, we have investigated how an agent determines optimal approach latency, but not how it controls withholding the response during this latency period, or how it invigorates the motor system to act as soon as the latency period has passed. Such motor control processes may possibly be described by inhibitory control theory. Crucially, BI is also necessary in many cognitive control tasks in which agents are explicitly instructed or incentivized to inhibit responses after receiving external stop signals, such as stop signal task, go/no go task or Stroop task (Logan, 1981; Logan et al., 1997). It has been proposed that these tasks generalize to more realistic scenarios in which stop signals are not externally imposed but have an internal, cognitive source. Such internal source could be learned by repeatedly experiencing external stop signals (Best, Lawrence, Logan, McLaren, & Verbruggen, 2016). Crucially, this investigation demonstrated that it is a stop goal that is learned, rather than an automatic or Pavlovian association of response inhibition with external sensory stop signals. This resonates with our account that anxiety-like BI also may be goal-directed. Individual differences in inhibitory control, that is, an ability to withhold a prepotent action, is suggested to relate to personality traits such as impulsivity (Logan et al., 1997). It may be possible that in our task, some variability in approach latencies stems from variance in motor processes. The execution of these processes in anxiety-like BI will be the topic of future research. We note that once a response is initiated, movement patterns do not differ between task conditions and are thus independent of threat probability or possible loss, as demonstrated previously in a joystick task (Bach, 2015).

To summarize, we find that anxiety-like BI appears under instrumental and possibly model-based control. This crucially constrains the search for the cognitive or computational algorithms governing this behavior. Furthermore, it also allows a more thorough understanding of the neural implementation underlying anxiety-like BI. The hippocampus appears a relevant structure for control of BI (Gray & McNaughton, 2000). Specifically, hippocampus lesions reduce BI in rodents (Bannerman et al., 2003; Bannerman et al., 2014; McHugh et al., 2004) and humans (Bach et al., 2014), power increases of ventral hippocampus theta oscillations are observed in rodent tests invoking BI (Adhikari et al., 2010; Gray & McNaughton, 2000), and anxiolytic drugs which reduce BI also reduce frequency (and power) of these oscillations (Gray & McNaughton, 2000). These descriptive observations await formalization. In this context, our cognitive model of BI control may enrich the investigation into underlying neurocomputational mechanisms.

Finally, BI is also a core feature of clinical anxiety states—as exemplified by worries that interfere with daily activities in generalized anxiety disorder (World Health Organization, 2004). Possible causes for this phenomenon are implausible assumptions on threat/reward correlations, suboptimal inference based on plausible priors, variations in instrumental mechanisms that evaluate action consequences, and also alterations in the execution of BI. Future work will examine to what extent such mechanisms may contribute to clinical conditions, and how to alleviate these.

## Supplementary Material

10.1037/xhp0000282.supp

## Figures and Tables

**Table 1 tbl1:** Approach/Avoidance Experiment 1: Statistical Analysis of Approach Latencies From a Linear Mixed Model With Random Intercepts, Both in a Full Model and Separately for Either of the Two Groups

Factor		*df*	*F*	*p*
Full model				
Group		1, 35748	35.72	<.001
Threat level		2, 35748	47.28	<.001
Potential loss		4, 35748	10.11	<.001
Group × Threat Level		2, 35748	49.31	<.001
Group × Potential Loss		4, 35748	4.69	<.001
Threat Level × Potential Loss		8, 35748	8.16	<.001
Group × Threat Level × Potential Loss		8, 35748	2.06	<.05
Group 1				
Threat level	omnibus	2, 15812	59.91	<.001
	linear	1, 15812	75.56	<.001
Potential loss	omnibus	4, 15812	5.97	<.001
	linear	1, 15812	<1	n.s.
Threat Level × Potential Loss	omnibus	8, 15812	4.14	<.001
	Linear × Linear	1, 15812	3.93	<.05
Group 2				
Threat level	omnibus	2, 15094	17.36	<.001
	linear	1, 15094	25.42	<.001
Potential loss	omnibus	4, 15094	2.75	<.05
	linear	1, 15094	2.14	n.s.
Threat Level × Potential Loss	omnibus	8, 15094	2.18	<.05
	Linear × Linear	1, 15094	<1	n.s.
*Note*. See Figure 2 for descriptive statistics.				

**Table 2 tbl2:** Approach/Avoidance Task 1 in Experiment 2: Statistical Analysis of Approach Latencies in a Linear Mixed Model With Random Intercepts

Factor	Effect	*df*	*F*	*p*
Threat level	omnibus	2, 10238	22.65	<.001
	linear	1, 10238	46.39	<.001
Potential loss	omnibus	4, 10238	8.99	<.001
	linear	1, 10238	25.20	<.001
Threat Level × Potential Loss	omnibus	8, 10238	1.52	n.s.
	Linear × Linear	1, 10238	6.67	=.01
*Note.* See Figure 3 for descriptive statistics.				

## References

[c1] Adhikari A., Topiwala M. A., & Gordon J. A. (2010). Synchronized activity between the ventral hippocampus and the medial prefrontal cortex during anxiety. Neuron, 65, 257–269. 10.1016/j.neuron.2009.12.002 20152131 PMC2822726

[c2] Amemori K., & Graybiel A. M. (2012). Localized microstimulation of primate pregenual cingulate cortex induces negative decision-making. Nature Neuroscience, 15, 776–785. 10.1038/nn.3088 22484571 PMC3369110

[c3] Aupperle R. L., Sullivan S., Melrose A. J., Paulus M. P., & Stein M. B. (2011). A reverse translational approach to quantify approach-avoidance conflict in humans. Behavioural Brain Research, 225, 455–463. 10.1016/j.bbr.2011.08.003 21843556 PMC3381365

[c4] Bach D. R. (2015). Anxiety-like behavioural inhibition is normative under environmental threat-reward correlations. PLoS Computational Biology, 11, e1004646 10.1371/journal.pcbi.1004646 26650585 PMC4674090

[c5] Bach D. R., Guitart-Masip M., Packard P. A., Miró J., Falip M., Fuentemilla L., & Dolan R. J. (2014). Human hippocampus arbitrates approach-avoidance conflict. Current Biology, 24, 541–547. 10.1016/j.cub.2014.01.046 24560572 PMC3969259

[c6] Bannerman D. M., Grubb M., Deacon R. M., Yee B. K., Feldon J., & Rawlins J. N. (2003). Ventral hippocampal lesions affect anxiety but not spatial learning. Behavioural Brain Research, 139, 197–213. 10.1016/S0166-4328(02)00268-1 12642189

[c7] Bannerman D. M., Sprengel R., Sanderson D. J., McHugh S. B., Rawlins J. N., Monyer H., & Seeburg P. H. (2014). Hippocampal synaptic plasticity, spatial memory and anxiety. Nature Reviews Neuroscience, 15, 181–192. 10.1038/nrn3677 24552786

[c8] Best M., Lawrence N. S., Logan G. D., McLaren I. P., & Verbruggen F. (2016). Should I stop or should I go? The role of associations and expectancies. Journal of Experimental Psychology: Human Perception and Performance, 42, 115–137. 10.1037/xhp0000116 26322688 PMC4685931

[c9] Burnham K. P., & Anderson D. R. (2004). Multimodel inference. Understanding AIC and BIC in model selection. Sociological Methods & Research, 33, 261–304. 10.1177/0049124104268644

[c10] Calhoon G. G., & Tye K. M. (2015). Resolving the neural circuits of anxiety. Nature Neuroscience, 18, 1394–1404. 10.1038/nn.4101 26404714 PMC7575249

[c11] Carver C. S., & White T. L. (1994). Behavioral inhibition, behavioral activation, and affective responses to impending reward and punishment: The BIS/BAS scales. Journal of Personality and Social Psychology, 67, 319–333. 10.1037/0022-3514.67.2.319

[c12] Corr P. J., & Cooper A. J. (2016). The Reinforcement Sensitivity Theory of Personality Questionnaire (RST-PQ): Development and validation. Psychological Assessment. Advance online publication 10.1037/pas0000273 26845224

[c13] Daw N. D., Niv Y., & Dayan P. (2005). Uncertainty-based competition between prefrontal and dorsolateral striatal systems for behavioral control. Nature Neuroscience, 8, 1704–1711. 10.1038/nn1560 16286932

[c14] Dayan P., & Balleine B. W. (2002). Reward, motivation, and reinforcement learning. Neuron, 36, 285–298. 10.1016/S0896-6273(02)00963-7 12383782

[c15] Dayan P., & Berridge K. C. (2014). Model-based and model-free Pavlovian reward learning: Revaluation, revision, and revelation. Cognitive, Affective & Behavioral Neuroscience, 14, 473–492. 10.3758/s13415-014-0277-8 PMC4074442 24647659

[c16] Dickinson A., & Balleine B. (1994). Motivational control of goal-directed action. Animal Learning & Behavior, 22, 1–18. 10.3758/BF03199951

[c17] Fonio E., Benjamini Y., & Golani I. (2009). Freedom of movement and the stability of its unfolding in free exploration of mice. Proceedings of the National Academy of Sciences of the United States of America, 106, 21335–21340. 10.1073/pnas.0812513106 19934049 PMC2795555

[c18] Fox N. A., & Pine D. S. (2012). Temperament and the emergence of anxiety disorders. Journal of the American Academy of Child and Adolescent Psychiatry, 51, 125–128. 10.1016/j.jaac.2011.10.006 22265356 PMC3619214

[c19] Friedman A., Homma D., Gibb L. G., Amemori K., Rubin S. J., Hood A. S., . . . Graybiel A. M. (2015). A corticostriatal path targeting striosomes controls decision-making under conflict. Cell, 161, 1320–1333. 10.1016/j.cell.2015.04.049 26027737 PMC4477966

[c20] Geller I., & Seifter J. (1960). A conflict procedure for the evaluation of drugs. Federation Proceedings, 19, 20.

[c21] Gigerenzer G., & Gaissmaier W. (2011). Heuristic decision making. Annual Review of Psychology, 62, 451–482. 10.1146/annurev-psych-120709-145346 21126183

[c22] Gray J. A. (1982). The neuropsychology of anxiety: An enquiry into the functions of the septohippocampal system. Oxford, UK: Oxford University Press.

[c23] Gray J. A., & McNaughton N. (2000). The neuropsychology of anxiety: An enquiry into the functions of the septohippocampal system (2nd ed.). Oxford, UK: Oxford University Press.

[c24] Hohle R. H. (1965). Inferred components of reaction-times as functions of foreperiod duration. Journal of Experimental Psychology, 69, 382–386. 10.1037/h0021740 14286308

[c25] Houlsby N. M., Huszár F., Ghassemi M. M., Orbán G., Wolpert D. M., & Lengyel M. (2013). Cognitive tomography reveals complex, task-independent mental representations. Current Biology, 23, 2169–2175. 10.1016/j.cub.2013.09.012 24354016 PMC3898796

[c26] Kalin N. H., & Shelton S. E. (1989). Defensive behaviors in infant rhesus monkeys: Environmental cues and neurochemical regulation. Science, 243, 1718–1721. 10.1126/science.2564702 2564702

[c27] Korn C. W., & Bach D. R. (2015). Maintaining homeostasis by decision-making. PLoS Computational Biology, 11, e1004301 10.1371/journal.pcbi.1004301 26024504 PMC4449003

[c28] Laux L., Glanzmann P., Schaffner P., & Spielberger C. D. (1981). Das State-Trait-Angstinventar [The State-Trait Anxiety Inventory]. Weinheim, Germany: Beltz.

[c29] Likhtik E., Stujenske J. M., Topiwala M. A., Harris A. Z., & Gordon J. A. (2014). Prefrontal entrainment of amygdala activity signals safety in learned fear and innate anxiety. Nature Neuroscience, 17, 106–113. 10.1038/nn.3582 24241397 PMC4035371

[c30] Logan G. D. (1981). Attention, automaticity, and the ability to stop a speeded choice response. Attention and performance IX, 205–222.

[c31] Logan G. D., Schachar R. J., & Tannock R. (1997). Impulsivity and inhibitory control. Psychological Science, 8, 60–64. 10.1111/j.1467-9280.1997.tb00545.x

[c32] Mackintosh N. J. (1975). Theory of attention—Variations in associability of stimuli with reinforcement. Psychological Review, 82, 276–298. 10.1037/h0076778

[c33] McHugh S. B., Deacon R. M., Rawlins J. N., & Bannerman D. M. (2004). Amygdala and ventral hippocampus contribute differentially to mechanisms of fear and anxiety. Behavioral Neuroscience, 118, 63–78. 10.1037/0735-7044.118.1.63 14979783

[c34] Miller N. E. (1944). Experimental studies of conflict In Hunt J. M. (Ed.), Personality and the behavior disorders (pp. 431–465). New York, NY: Ronald.

[c35] Pearce J. M., & Hall G. (1980). A model for Pavlovian learning: Variations in the effectiveness of conditioned but not of unconditioned stimuli. Psychological Review, 87, 532–552. 10.1037/0033-295X.87.6.532 7443916

[c36] Pellow S., Chopin P., File S. E., & Briley M. (1985). Validation of open:closed arm entries in an elevated plus-maze as a measure of anxiety in the rat. Journal of Neuroscience Methods, 14, 149–167. 10.1016/0165-0270(85)90031-7 2864480

[c37] Penny W. D., Stephan K. E., Mechelli A., & Friston K. J. (2004). Comparing dynamic causal models. NeuroImage, 22, 1157–1172. 10.1016/j.neuroimage.2004.03.026 15219588

[c38] Prevedello J. A., Dickman C. R., Vieira M. V., & Vieira E. M. (2013). Population responses of small mammals to food supply and predators: A global meta-analysis. Journal of Animal Ecology, 82, 927–936. 10.1111/1365-2656.12072 23560951

[c39] Raftery A. E. (1995). Bayesian model selection in social research. Sociological Methodology, 25, 111–163. 10.2307/271063

[c40] Rescorla R. A., & Wagner A. R. (1972). A theory of Pavlovian conditioning: Variations in the effectiveness of reinforcement and nonreinforcement In Black A. H. & Prokasy W. F. (Eds.), Classical conditioning II: Current research and theory (pp. 64–99). New York, NY: Appleton-Century-Crofts.

[c41] Rodgers R. J., Cao B. J., Dalvi A., & Holmes A. (1997). Animal models of anxiety: An ethological perspective. Brazilian Journal of Medical and Biological Research, 30, 289–304. 10.1590/S0100-879X1997000300002 9246227

[c42] Schwartz B., & Williams D. R. (1972). The role of the response-reinforcer contingency in negative automaintenance. Journal of the Experimental Analysis of Behavior, 17, 351–357. 10.1901/jeab.1972.17-351 16811590 PMC1333910

[c43] Sofaer H. R., Sillett T. S., Peluc S. I., Morrison S. A., & Ghalambor C. K. (2013). Differential effects of food availability and nest predation risk on avian reproductive strategies. Behavioral Ecology, 24, 698–707. 10.1093/beheco/ars212

[c44] Stephan K. E., Penny W. D., Daunizeau J., Moran R. J., & Friston K. J. (2009). Bayesian model selection for group studies. NeuroImage, 46, 1004–1017. 10.1016/j.neuroimage.2009.03.025 19306932 PMC2703732

[c45] Sutton R. S., & Barto A. G. (1998). Reinforcement learning: An introduction. Cambridge, MA: MIT Press.

[c46] Vogel J. R., Beer B., & Clody D. E. (1971). Simple and reliable conflict procedure for testing anti-anxiety agents. Psychopharmacologia, 21, 1–7. 5105868 10.1007/BF00403989

[c47] World Health Organization (2004). International statistical classification of diseases and health related problems (10th revision) ICD-10. Geneva, Switzerland: Author.

