# Supplementary material for: The Cognitive Architecture of Anxiety-Like Behavioral Inhibition
Source: J Exp Psychol Hum Percept Perform. 2016 Oct 31;43(1):18–29. doi: 10.1037/xhp0000282 (PMC5178866; doi:10.1037/xhp0000282)

1. Supplemental methods: Derivation of the null distribution in the safe predator exposure task

Table 1: List of variables used in the derivation of the null distribution.

Variable	Explanation
T	Time of key press, expressed with respect to the previous token appearance at t_0 (random variable)
t_0	Time of previous token appearance
T_1	Time of token disappearance (random variable)
T_2	Time of next token appearance (random variable)
t	Observed time of key press
t_c	Constant minimum waiting time between token disappearance and start of next token
t_{max}	Maximum appearance and disappearance time
λ	Parameter of the exponential distributions governing token appearance and disappearance
$O \in \{0, 1\}$	Binary random variable for the observation of any key press after a token appearance

The appearance time of the next token is the sum of two exponential distributions with the same mean $1/\lambda$, plus a constant t_c:

$$T_2 = (T_2 - T_1) + (T_1 - t_c) + t_c$$

$$(T_2 - T_1) \sim Exp(\lambda)$$

$$(T_1 - t_c) \sim Exp(\lambda)$$

$$\Rightarrow (T_2 - t_c) \sim Gamma(2, 1/\lambda).$$

This is an Erlang distribution with rate parameter λ, shifted by t_c. Both exponential distributions are truncated at t_{max}, i. e. all values larger than t_{max} are set to t_{max}, and this renders the distribution analytically intractable outside the interval $t \in [0, t_c + t_{max}]$. We therefore restricted our analysis to this interval, which included 99.8% of the actually occurring key presses after a token. Within this interval, we can use an analytical expression for the CDF of a gamma distribution, to derive the pdf of the token appearance, where we omit the normalising constant:

$$p_{T_2>t}(t) \propto \begin{cases} 0 & t < 0 \\ 1 & 0 < t < t_c \\ (1 - \gamma(2, \lambda(t - t_c))) & t_c < t < (t_{max} + t_c) \\ g(t) & t > (t_{max} + t_c) \end{cases}.$$

To compute a Kolmogoroff-Smirnoff test in R, we used an analytical form for the cdf of this pdf, denoted $F_{T_2>t}$. We recall a standard result for integration of the incomplete gamma function:

$$\int x^{b-1}\gamma\left(s,x\right)dx=\frac{1}{b}\left(x^{b}\gamma\left(s,x\right)+\Gamma\left(s+b,x\right)\right). \tag{1}$$

Then the cdf is:

$$F_{T_2>t}\left(t\right)\propto\int_0^t dy\begin{cases}0 & t<0\\ 1 & 0<t<t_c\\ \left(1-\gamma\left(2,\lambda\left(y-t_c\right)\right)\right) & t_c<t<\left(t_{max}+t_c\right)\\ G\left(t\right) & t>\left(t_{max}+t_c\right)\end{cases}$$

$$=\begin{cases}0 & t<0\\ t & 0<t<t_c\\ F_{T_2>t}\left(t_c\right)+\int_{t_c}^t dy\left(1-\gamma\left(2,\lambda\left(y-t_c\right)\right)\right) & t_c<t<\left(t_{max}+t_c\right)\\ G\left(t\right) & t>\left(t_{max}+t_c\right)\end{cases}$$

$$=\begin{cases}0 & t<0\\ t & 0<t<t_c\\ F_{T_2>t}\left(t_c\right)+\lambda^{-1}\int_0^{\lambda(t-t_c)} du\left(1-\gamma\left(2,u\right)\right) & t_c<t<\left(t_{max}+t_c\right)\\ G\left(t\right) & t>\left(t_{max}+t_c\right)\end{cases}$$

$$=\begin{cases}0 & t<0\\ t & 0<t<t_c\\ F_{T_2>t}\left(t_c\right)+\lambda^{-1}\left[u-u\gamma\left(2,u\right)-\Gamma\left(3,u\right)\right]_0^{\lambda(t-t_c)} & t_c<t<\left(t_{max}+t_c\right)\\ G\left(t\right) & t>\left(t_{max}+t_c\right)\end{cases},$$

where we used eq. (1) with $b=1$. We have: $\Gamma\left(s,0\right)=\Gamma\left(s\right)=\left(s-1\right)!$, for $s\in\mathbb{N}$. Hence

$$F_{T_2>t}\left(t\right)=\begin{cases}0 & t<0\\ t & 0<t<t_c\\ t+2\lambda^{-1}-\left(t-t_c\right)\gamma\left(2,\lambda\left(t-t_c\right)\right)-\lambda^{-1}\Gamma\left(3,\lambda\left(t-t_c\right)\right) & t_c<t<\left(t_{max}+t_c\right)\\ G\left(t\right) & t>\left(t_{max}+t_c\right)\end{cases}$$

$$=\begin{cases}0 & t<0\\ t & 0<t<t_c\\ t+2\lambda^{-1}-\left(t-t_c\right)\left(1-\Gamma\left(2,\lambda\left(t-t_c\right)\right)\right)-\lambda^{-1}\Gamma\left(3,\lambda\left(t-t_c\right)\right) & t_c<t<\left(t_{max}+t_c\right)\\ G\left(t\right) & t>\left(t_{max}+t_c\right)\end{cases}. \tag{2}$$

Where we have replaced the lower with the upper incomplete gamma function for use in R.

2. Supplemental results

Table 2: Experiment 1, influence of time on approach latency. Results from a 2×3×5 + 2 (group)×6 (task block) Linear Mixed Effects Model on approach latency.

	**Approach latency**		
	df	*F*	*p*
Group	1, 35738	35.76	< 1e-8
Threat level	2, 35738	47.90	< 1e-20
Potential loss	4, 35738	10.64	< 1e-7
Threat level x potential loss	8, 35738	8.43	< 1e-10
Group x threat level	2, 35738	49.17	< 1e-21
Group x potential loss	4, 35738	4.50	< .001
Group x threat level x potential loss	8, 35738	2.02	< .05
Experiment block	5, 35738	27.67	< 1e-27
Group x block	5, 35738	4.33	< .001

Figure 1: Experiment 1, influence of time on approach latency. Apporach latencies are estimated for the 6 blocks of the experiment from a linear mixed model with random intercepts .

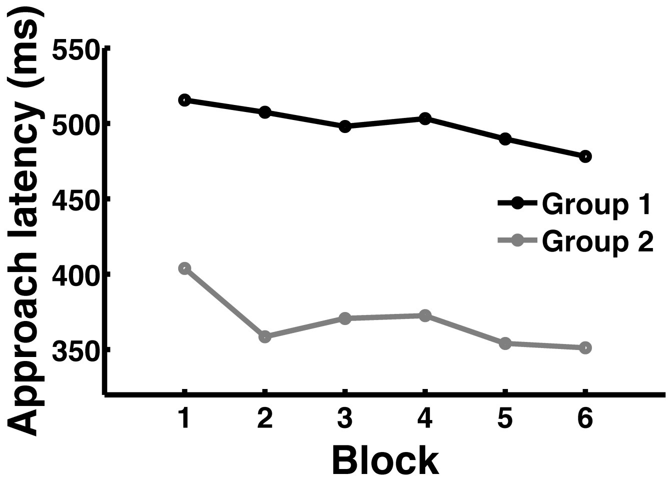

Table 3: Experiment 1, ancillary measures. Results from a 3×6 or 2×5 or 2×3×5 (group comparison) Linear Mixed Effects Model on choice, return latencies, and flight latency. No exact p-values can be computed for choice data (see methods).

	df	*F*	*df*	*F*	*p*
**Group 1**	**Choice**		**Return latency**		
Threat level	2, 17687	6.83	2, 12614	10.14	$<$ .0001
Potential loss	5, 17687	802.10	4, 12614	10.58	$<$ 1e-7
Threat level x potential loss	10, 17687	2.06	8, 12614	$<$ 1	n. s.
**Group 2**	**Choice**		**Return latency (step 1)**		
Threat level	2, 16769	12.08	2, 13054	$<$ 1	n. s.
Potential loss	5, 16769	862.46	4, 13054	9.79	$<$ 1e-7
Threat level x potential loss	10, 16769	2.18	8, 13054	1.29	n. s.
			**Return latency (step 2)**		
Threat level			2, 9759	15.20	$<$ 1e-6
Potential loss			4, 9759	1.01	n. s.
Threat level x potential loss			8, 9759	1.29	n. s.
			**Flight latency**		
Threat level			2, 5873	2.42	n. s.
Potential loss			4, 5873	7.16	$<$ 1e-5
Threat level x potential loss			8, 5873	2.41	$<$ .05
**Group comparison**	**Choice**				
Group	1, 34455	$>$ 1			
Threat level	2, 34455	552.34			
Potential loss	5, 34455	6804.10			
Threat level x potential loss	10, 34455	31.71			
Group x threat level	2, 34455	32.11			
Group x potential loss	5, 34455	13.02			
Group x threat level x potential loss	10, 34455	2.10			

Figure 2: Experiments 1/2, ancillary measures. Escape latency (experiment 1, group 2), return latencies, and correctness of response (experiment 2). In experiment 1, group 2, two movements were required to return to the safe place after token collection; hence we report two return latencies. As the data are unbalanced, mean approach latencies were estimated in a linear mixed effects model with random intercepts.

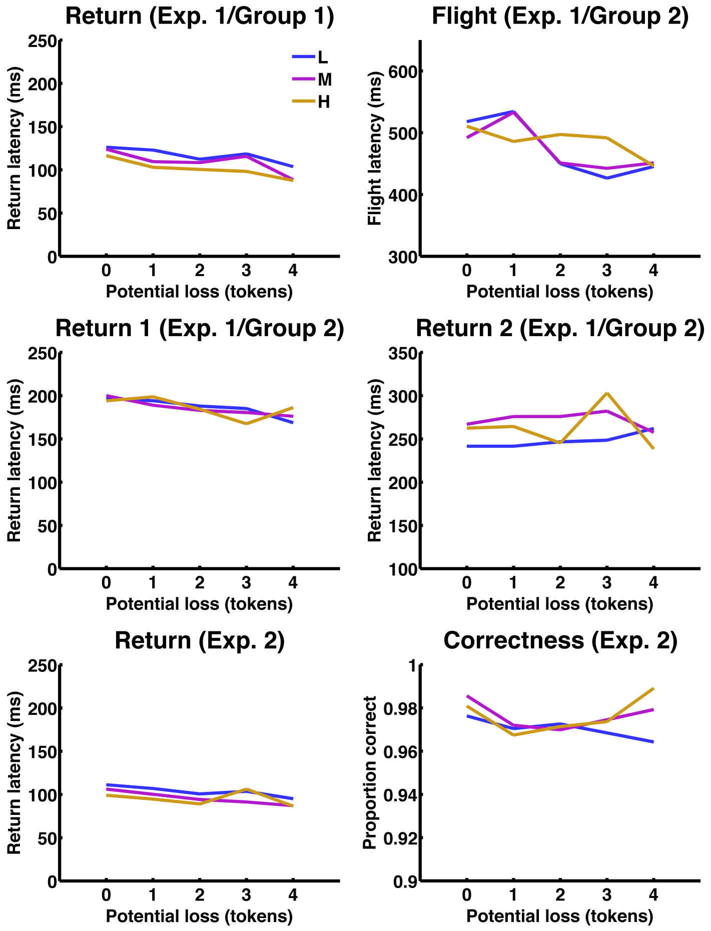

Figure 3: Experiment 2, individual participants' RT distributions in the safe predator exposure task. Blue lines: null distribution. Red lines: participant-specific fit to combined model.

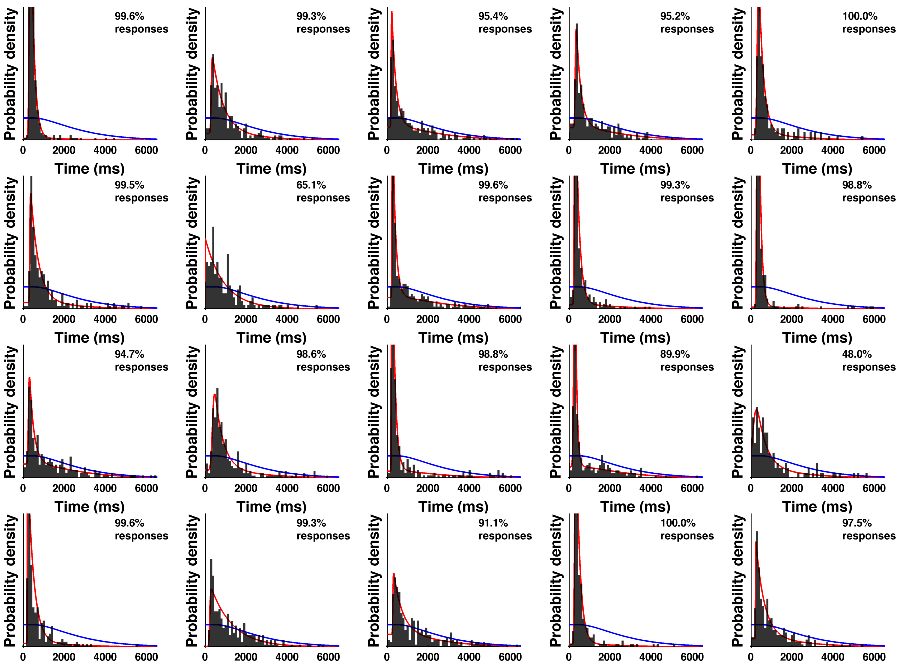

Table 4: Experiment 2, approach/avoidance task 1. Results from a 3×5 or 2×4 Linear Mixed Effects Model on choice, return latency, and correctness of response. No exact p-values can be computed for choice data (see methods).

	**Choice**		**Return latency**			**Correctness**		
	df	*F*	*df*	*F*	*p*	*df*	*F*	*p*
Threat level	2, 11993	3.90	2, 8649	16.67	< 1e-7	2, 10329	< 1	n. s.
Threat level: linear			1, 8649	10.28	< .005			
Potential loss	5, 11993	572.53	4, 8649	10.31	< 1e-7	4, 10329	2.93	< .05
Potential loss: linear			1, 8649	13.11	< .001			
Threat level × potential loss	8, 11993	< 1	8, 8649	1.26	n. s.	8, 10329	< 1	n. s.
Interaction: linear × linear			1, 10238	1.04	n. s.			

Figure 4: Experiments 1/2, debriefing results. Participants were asked to rate, on a scale from 0-100%, how likely it was the the predator caught them if they were outside the safe place. On the x-axis we show the actual catch rates, which depended on participants' movement latencies.

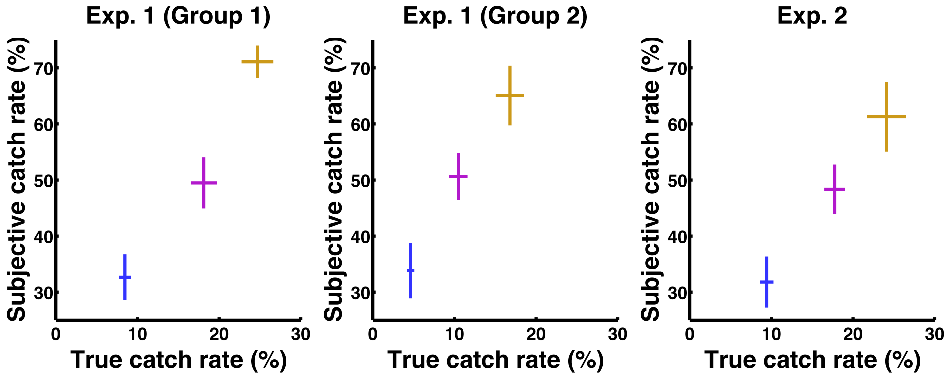

Supplement: Supplementary file 1 [file BACH_AAA2_SI_004.pdf]
